# Antibodies to probe endogenous G protein-coupled receptor heteromer expression, regulation, and function

**DOI:** 10.3389/fphar.2014.00268

**Published:** 2014-12-03

**Authors:** Ivone Gomes, Achla Gupta, Ittai Bushlin, Lakshmi A. Devi

**Affiliations:** ^1^Department of Pharmacology and Systems Therapeutics, Icahn School of Medicine at Mount SinaiNew York, NY, USA; ^2^The Friedman Brain Institute, Icahn School of Medicine at Mount SinaiNew York, NY, USA

**Keywords:** G protein-coupled receptor, dimerization, heteromerization, opioid, cannabinoid, angiotensin

## Abstract

Over the last decade an increasing number of studies have focused on the ability of G protein-coupled receptors to form heteromers and explored how receptor heteromerization modulates the binding, signaling and trafficking properties of individual receptors. Most of these studies were carried out in heterologous cells expressing epitope tagged receptors. Very little information is available about the *in vivo* physiological role of G protein-coupled receptor heteromers due to a lack of tools to detect their presence in endogenous tissue. Recent advances such as the generation of mouse models expressing fluorescently labeled receptors, of TAT based peptides that can disrupt a given heteromer pair, or of heteromer-selective antibodies that recognize the heteromer in endogenous tissue have begun to elucidate the physiological and pathological roles of receptor heteromers. In this review we have focused on heteromer-selective antibodies and describe how a subtractive immunization strategy can be successfully used to generate antibodies that selectively recognize a desired heteromer pair. We also describe the uses of these antibodies to detect the presence of heteromers, to study their properties in endogenous tissues, and to monitor changes in heteromer levels under pathological conditions. Together, these findings suggest that G protein-coupled receptor heteromers represent unique targets for the development of drugs with reduced side-effects.

## Introduction

Since the first report showing that metabotropic GABA_B_ receptors, members of the family C of G protein-coupled receptors (GPCRs), form constitutive heteromers (White et al., 1998; Kuner et al., 1999; Pin et al., 2009) an increasing number of studies have provided evidence suggesting that other GPCRs, particularly those belonging to family A, also heteromerize (Albizu et al., 2010; Gomes et al., 2013a; Hiller et al., 2013; Szafran et al., 2013). However, most of the studies reporting GPCR heteromerization were carried out in heterologous cells co-expressing differentially epitope tagged recombinant receptors. Concerns that heteromerization in heterologous cells could be due to over-expression of individual receptors and that the unique signaling reported for a given heteromer is due to receptor cross-talk via downstream signaling rather than direct receptor-receptor interactions led investigators in the field to propose a set of criteria to be fulfilled in order to consider that a GPCR pair forms an heteromer in endogenous tissue (Ferre et al., 2009): (i) both receptors can be detected in the same subcellular compartment in a cell; (ii) close proximity between the two receptors for direct interactions can be demonstrated through the use of either proximity ligation assays, ligand-based FRET, or heteromer-selective probes such as antibodies only in wild-type tissue; (iii) the receptors can be co-immunoprecipitated from wild-type but not from tissue lacking one of the receptors; (iv) the heteromer pair exhibits a “biochemical fingerprint” in wild-type tissue that matches that seen in heterologous cells co-expressing both receptors but not cells expressing only one of the receptors; and (v) heteromer formation can be disrupted by agents such as TAT peptides and this leads to alterations in the “biochemical fingerprint” to one that resembles that of individual receptor protomers (Ferre et al., 2009).

In order to detect and map the presence of a GPCR heteromer in endogenous tissue, sensitive and selective tools are needed. Such tools could help not only to monitor heteromer levels under physiological and pathological conditions but also to tease apart the contribution of receptor homomers and heteromers to a given physiological response. In order to address this need our laboratory undertook the challenge to generate monoclonal antibodies that selectively recognize a given heteromer pair. Since monoclonal antibodies recognize a single epitope and are highly specific, they would not only facilitate detection of the targeted heteromer in endogenous tissue but would also permit studies to elucidate the contribution of the heteromer to signaling in tissues/membranes expressing both receptors. In general it is easy to generate antibodies to immunodominant and abundant epitopes; however this task is more challenging when using epitopes that are likely to be rare or less immunodominant. This would be the case with “heteromer-selective” epitopes where very little is known about the “heteromer” interface. We therefore decided to use a subtractive immunization strategy to improve our changes of raising such antibodies. This strategy has been successfully used in the cancer field to generate monoclonal antibodies that can specifically block metastasis but not proliferation of cancer cells (Brooks et al., 1993), antibodies that can discriminate proteins that have a similar sequence (Sleister and Rao, 2002), or antibodies that could be used as diagnostic tools in certain types of cancer (Trefzer et al., 2000; Yasumoto et al., 2012). In this review we describe the strategy used to generate and characterize antibodies selective to either μ OR-δ OR, δ OR-κ OR, δ OR-CB1R, and AT1R-CB1R heteromers (Table 1).

**Table 1:** **Genertion and characterization of heteromer-selective antibodies and their potential therapeutic applications**.

**Heteromer pair detected**	**Antibodies generation scheme**	**Heteromer selectivity tested using**	**Properties**	**Potential therapeutic applications**	**Ref**.
μ OR-δ OR	Tolerization: HEK293 membranes	(i) Membranes from HEK293 cells alone, cells expressing either μ OR, δ OR or μ OR-δ OR;	(a) Detects changes in heteromer levels in endogenous tissue;	Tolerance	Gupta et al., 2010
	Immunization: HEK293 membranes expressing μ OR-δ OR	(ii) Membranes from cells expressing either μ OR or δ OR in combination with other GPCRs;	(b) Blocks heteromer-mediated binding & signaling		
		(iii) Membranes from cells expressing different ratios of μ OR and δ OR;			
		(iv) Membranes from cells expressing chimeric μ OR-δ OR constructs;			
		(v) Membranes from wild-type, μ OR k/o or δ OR k/o tissue			
κ OR-δ OR	Tolerization: HEK293 membranes	(i) Membranes from HEK293 cells alone, cells expressing either κ OR, δ OR, or κ OR-δ OR;	Potentiates DPDPE- mediated antinocipeption during thermal allodynia	Antinociception	Berg et al., 2012
	Immunization: HEK293 membranes expressing κ OR-δ OR	(ii) HEK293 cells expressing either κ OR, δ OR, CB1R, μ OR-δ OR or κ OR-δ OR;			
		(iii) Neuro2A cells expressing either CB1R-AT1R, CB1R-CBR2, CB1R-μ OR, CB1R-δ OR; and CB1R-κ OR			
δ OR-CB1R	Tolerization: Neuro2A* membranes	(i) Membranes from Neuro2A* cells alone or in combination with either δ OR, μ OR, κ OR, CB2R or AT1R;	(a) Detects changes in heteromer levels in endogenous tissue;	Neuropathic pain	Bushlin et al., 2012
	Immunization: Neuro2A* membranes expressing δ OR	(ii) Membranes from HEK293 cells expressing either δ OR alone or in combination with either μ OR or κ OR;	(b) Blocks CB1R agonist-mediated increases in δ OR activity		
		(iii) Cortical membranes from either wild-type, CB1R k/o or δ OR mice			
CB1R-AT1R	Tolerization: Neuro2A* membranes	(i) Membranes from HEK293 expressing either CB1R, AT1R or CB1R-AT1R;	(a) Detects changes in heteromer levels in activated HSCs;	Treatment of liver fibrosis	Rozenfeld et al., 2011
	Immunization: Neuro2A* membranes expressing AT1R	(ii) HEK293 cells expressing different ratios of CB1R and AT1R;	(b) Blocks angiotensin II-mediated signaling only in cells co-expressing CB1R-AT1R;		
		(iii) HEK293 cells expressing either CB1R, AT1R, or CB1R-CB2R, CB1R-δ OR, CB1R-μ OR, CB1R-κ OR, μ OR-δ OR or κ OR- δ OR	(c) Decreases secretion of fibrogenic proteins from activated HSCs		
		(iv) Membranes from Neuro2A* cells alone or in combination with AT1R (CB1R-AT1R cells) and CB1R-AT1R cells where CB1R levels were reduced by expression of RNAi			

^*^Neuro2A cells endogenously express CB1R.

AT1R, angiotensin type 1 receptor; CB1R, cannabinoid type 1 receptor; CB2R, cannabinoid type 2 receptor; ELISA, enzyme-linked immunosorbent assay; HSCs, hepatic stellate cells; IF, immunofluorescence; IHC, immunohistochemistry; IP, immunoprecipitation; k/o, knockout; n.d., not determined.

## Generation of heteromer-selective antibodies using a subtractive immunization strategy

An important requirement to generate antibodies that can selectively recognize a given GPCR heteromer is the immunogen. An ideal immunogen would be a synthetic peptide that mimics the heteromeric region between two GPCRs since the latter would be distinct and unique compared to the homomeric regions. However, not much is known about the heteromer interface or a unique region shared by heteromers. Hence we used membranes from cells expressing the heteromer pair of interest as the immunogen. Given the likelihood that the heteromeric epitopes would be of very low abundance and of low immunogenicity, thereby preventing their detection by antibody producing cells, direct immunization with such membranes would have a low probability of successfully generating heteromer-selective antibodies. Therefore in order to improve our chances of generating heteromer-selective antibodies we used a subtractive immunization strategy (Salata et al., 1992; Sleister and Rao, 2001, 2002) that involves two major steps: (i) tolerization of mice to unwanted epitopes, and (ii) immunization with membranes expressing the heteromer pair of interest. Tolerization to unwanted epitopes can be achieved by immunizing mice with an emulsion of membranes from cells used to express the heteromer pair in combination with complete Freund's adjuvant (Gomes et al., 2013b); these cells can be CHO or HEK-293 cells that are usually used in GPCR co-expression studies as well as cells that endogenously express one of the receptor protomers (Gomes et al., 2013b). The mice are then treated for the next 3 days with cyclophosphamide to kill activated antibody producing cells (Gomes et al., 2013b). Every 15 days mice are administered with booster injections comprised of membrane emulsions in Freund's incomplete adjuvant followed by the 3 day treatment with cyclophosphamide (Gomes et al., 2013b). Booster injections are repeated until a consistently low titer is observed by ELISA with the membranes used for the tolerization step (Gomes et al., 2013b). Once animals are tolerized they are immunized with membranes expressing the heteromer pair of interest (Gomes et al., 2013b). Booster injections are repeated until a high titer is obtained by ELISA (using membranes that co-express both receptors). Animals are killed and individual spleens used to generate monoclonal antibodies using standard protocols (Gomes et al., 2013b). Once monoclonal antibodies are obtained, individual clones are tested for heteromer selectivity by ELISA, immunofluorescence or Western blot analysis using (i) cells that express individual receptors; (ii) cells that co-express both of the protomers of interest; (iii) cells that co-express one of the receptor protomers with a different partner GPCR; and (iv) tissues from wild-type and from animals lacking each of the receptor protomers (Gomes et al., 2013b). An antibody is considered to be heteromer-selective only if it gives a signal with cells or tissues co-expressing both of the protomers of interest. It is to be noted that heteromer selectivity may be observed with one screening procedure such as ELISA but not with another such as Western blotting or immunofluorescence since either heat denaturation of membrane proteins (as in the case of Western blot analysis) and/or tissue fixation (in the case of immunofluorescence studies) could mask the epitope identified by the antibody. Thus one needs to be careful about selecting the screening technique to allow for detection of the antigen under the assay of choice. Using this subtractive immunization strategy we successfully generated antibodies selective for either μ OR-δ OR, δ OR-κ OR, δ OR-CB1R, or AT1R-CB1R heteromers (Gupta et al., 2010; Rozenfeld et al., 2011; Berg et al., 2012; Bushlin et al., 2012). In the following sections we describe these heteromer pairs and the studies carried out using heteromer selective antibodies.

## μOR-δOR heteromerization

A number of early studies proposed heteromerization between μ OR and δ OR based on interactions between these receptors. For example, pharmacological studies showed that morphine (a μ OR agonist) shifted competitive radiolabeled leucine-enkephalin displacement curves by unlabeled leucine-enkephalin (a δ OR agonist) into non-competitive curves (Rothman and Westfall, 1982). In addition behavioral studies showed that δ OR agonists (endogenous peptides or synthetic agonists) could potentiate μ OR-mediated antinociception while potent δ OR antagonists attenuated not only morphine-mediated antinociception but also the development of tolerance to this drug (reviewed in Fujita et al., 2014a). Furthermore studies showed that chronic treatment with morphine increases surface expression of δ OR in either cultured cortical or dorsal root ganglion neurons and in the dorsal horn of the spinal cord of wild-type but not in mice lacking μ OR (Cahill et al., 2001; Morinville et al., 2003; Gendron et al., 2006). Studies with animals lacking either μ OR or δ OR further support interactions between these receptors. These studies indicate that δ OR-mediated antinociception requires the presence of functional μ OR (Matthes et al., 1996, 1998) and that δ OR contributes to the development of tolerance to morphine (Zhu et al., 1999). The latter observation is also supported by studies using antisense oligonucleotides to decrease δ OR expression in the brain (Sanchez-Blazquez et al., 1997). In addition, it has been reported that treatment with a selective δ OR antagonist, naltriben, reduces the rewarding effects of morphine as measured using the morphine conditioned place preference test and this is accompanied by increases in δ OR levels at the post-synaptic density fraction (Billa et al., 2010). Taken together these studies suggested receptor-receptor interactions between μ OR and δ OR.

A major requirement for two receptors to directly interact with each other is that they be localized not only to the same cell but also to the same subcellular compartment. Early evidence for the presence of μ OR and δ OR in the same cell came from electrophysiological and radiolabeled binding studies using either neurons or neuroblastoma cell lines (Egan and North, 1981; Zieglgansberger et al., 1982; Yu et al., 1986; Kazmi and Mishra, 1987; Baumhaker et al., 1993; Palazzi et al., 1996). In addition, a number of immunohistochemical studies showed that both receptors were present in the same subcellular compartment in the brain and spinal cord by using receptor-selective antibodies (Arvidsson et al., 1995; Cheng et al., 1997; Wang and Pickel, 2001). Controversy arose about the co-localization of μ OR and δ OR in the dorsal root ganglions of the spinal cord primarily due to data with mice with a knockin of eGFP-tagged δ OR that showed that both receptors were segregated from each other (<5% neurons showed receptor colocalization) with μ OR being expressed in small peptidergic neurons where it was involved in inhibition of pain induced by noxious heat while δ OR was expressed in medium-sized non-peptidergic and large myelinated neurons where it was involved in inhibition of pain induced by mechanical stimuli (Scherrer et al., 2009). However, previous and recent observations questioned the lack of μ OR and δ OR colocalization. For example, support for colocalization came from (i) studies using either immunogold electron microscopy (Cheng et al., 1997), single-cell PCR, *in situ* hybridization or immunostaining to demonstrate the presence of μ OR and δ OR in small peptidergic DRG neurons (Wang et al., 2010); (ii) studies showing that *myc*-tagged δ OR is present in CGRP-containing large dense core vesicles while eGFP-tagged δ OR is present at the cell surface when expressed in small DRGs (Zhang and Bao, 2012) suggesting that the C-terminal GFP might affect receptor trafficking; (iii) studies treating peptidergic nociceptors expressing μ OR and δ OR with selective agonists that prevent substance P release induced by formalin or capsaicin treatment and this could be blocked by receptor selective antagonists (Beaudry et al., 2011); (iv) studies using electrophysiological recordings from a wide range of neurons in the spinal trigeminal nucleus of anesthetized animals showing that activation of either μ OR or δ OR relieves both thermal- or mechanical induced pain with same potency (Normandin et al., 2013); and (v) studies showing co-localization of μ OR and δ OR in the plasma membrane of a small population of CGRP-containing neurons in eGFP-tagged δ OR knockin mice (Bardoni et al., 2014). Additional support for co-localization comes from mice expressing eGFP-tagged δ OR and mCherry-tagged μ OR. These mice show that ~40% of eGFP-tagged δ OR positive and ~30% of mCherry-tagged μ OR positive DRGs co-express the two receptors (Erbs et al., 2014). In addition, these mice show colocalization of μ OR and δ OR in neurocircuits involved in survival, pain regulation, as well as food intake, water consumption and sexual behavior (Erbs et al., 2014). In the hippocampus co-expression of eGFP-tagged δ OR and mCherry-tagged μ OR is detected in GABAergic interneurons and formation of μ OR-δ OR interacting complexes was demonstrated by co-immunoprecipitation studies (Erbs et al., 2014). Taken together these results demonstrate substantial co-localization of μ OR or δ OR in the brain and spinal cord.

In order to detect the presence of μ OR-δ OR heteromers in endogenous tissue our laboratory generated heteromer-selective antibodies (Table 1) using a subtractive immunization strategy (Gupta et al., 2010). ELISA with these antibodies show that they detect an epitope present only in cells co-expressing μ OR and δ OR and not in cells expressing individual receptors or co-expressing either μ OR or δ OR in combination with other GPCRs (Gupta et al., 2010). Moreover, the signal obtained in ELISA is reduced when the antibodies are pre-incubated with membranes co-expressing μ OR and δ OR but not with membranes expressing individual receptors (Gupta et al., 2010). In addition, these antibodies recognize an epitope present only in membranes from wild-type mice but not from mice lacking either μ OR or δ OR (Gupta et al., 2010). Furthermore, the heteromer-selective antibodies showed better recognition of co-expressed wild-type receptors compared to co-expressed chimeric receptors where regions of μ OR were substituted with δ OR and *vice-versa* (Gupta et al., 2010). Taken together these results indicate that the antibodies selectively recognize the μ OR-δ OR heteromer.

**Figure 1:**
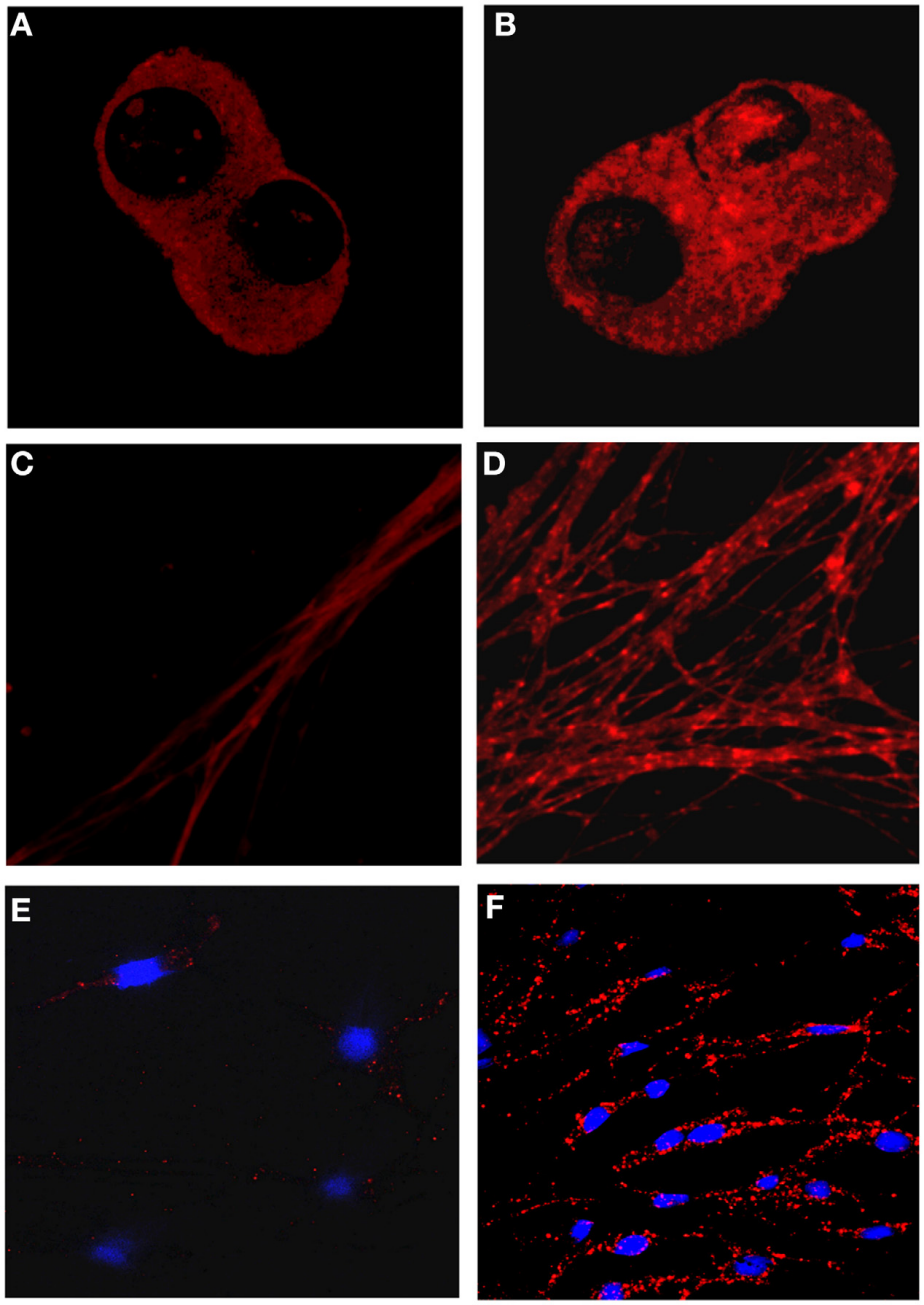
**Detection of μOR-δOR heteromers in primary dorsal root ganglion neurons using heteromer-selective antibodies**. **(A–D)** Primary dorsal root ganglion neurons (DRGs) from embryonic rats were treated without **(A,C)** or with 10 μ M morphine **(B,D)** for 48 h. μ OR-δ OR heteromers were visualized in the cell bodies **(A,B)** or in dendrites **(B,D)** using heteromer-selective antibodies (red). **(E,F)** Primary DRGs from adult rats were treated without **(E)** or with 10 μ M morphine **(F)** for 48 h and μ OR-δ OR heteromers visualized using heteromer-selective antibodies (red). Morphine treatment increases μ OR-δ OR heteromer levels. Blue color represents nuclear DAPI staining.

The μ OR-δ OR heteromer-selective antibodies can be used for immunohistochemical studies to detect the presence of these heteromers in endogenous tissue or primary DRG cultures (Gupta et al., 2010). An interesting finding with these antibodies is that chronic treatment with escalating doses of morphine under conditions that lead to the development of antinociceptive tolerance leads to an increase in μ OR-δ OR heteromers in select brain regions from wild-type but not from mice lacking either μ OR or δ OR (Gupta et al., 2010). These regions include the medial nucleus of the trapezoid body (MNTB), an auditory relay nucleus and the rostral ventral medulla (RVM), a key relay nucleus involved in pain perception (Gupta et al., 2010). Similar increases in μ OR-δ OR heteromers were also observed in the cell bodies and dendrites of primary DRG neurons following 48 h treatment with morphine (Figure 1). More recently μ OR-δ OR heteromer-selective antibodies were used to detect the presence of these heteromers in ileal tissue (Fujita et al., 2014b).

Another criteria that a μ OR and δ OR heteromer has to fulfill is that both receptor protomers have to be in close enough proximity to directly interact. Co-immunoprecipitation studies using either antibodies to epitope tags or to endogenous receptors show that μ OR and δ OR form interacting complexes only in spinal cord membranes from wild-type (but not from mice lacking one of the receptors) as well as in cells co-expressing both receptors (George et al., 2000; Gomes et al., 2000, 2004). In addition we find that the μ OR-δ OR heteromer-selective antibodies can immunoprecipitate the heteromer from primary dorsal root ganglion (DRG) neurons as well as from cells co-expressing both receptors (Gupta et al., 2010). That μ OR and δ OR are in close proximity to directly interact was further supported by proximity based assays showing that the two receptors are <100Å in live cells co-expressing both receptors (Gomes et al., 2004; Hasbi et al., 2007).

A third criteria that the μ OR-δ OR heteromer has to fulfill is that it exhibits a unique “biochemical fingerprint” that is seen only in cells/tissues expressing both receptors. The “biochemical fingerprint” for μ OR-δ OR heteromers consists of changes in ligand binding and signaling properties. These include (i) the binding affinity of selective synthetic agonists is decreased while that of endogenous peptidic agonists is increased (George et al., 2000); (ii) occupancy of a receptor protomer allosterically modulates the binding and signaling profile of the partner protomer (Gomes et al., 2000, 2004, 2011); (iii) the μ OR-δ OR heteromer signals via either pertussis toxin insensitive Gαz (George et al., 2000; Fan et al., 2005; Hasbi et al., 2007), pertussis toxin sensitive Ca^+2^ signaling (Charles et al., 2003), or β-arrestin2 (Rozenfeld and Devi, 2007) compared to individual receptor homomers that signal via pertussis sensitive Gαi. A related point supporting receptor-receptor interactions is changes in maturation, endocytosis and degradation. For example, a study showed that co-expression of μ OR and δ OR leads to retention of the heteromer in the Golgi and that increased cell surface expression of μ OR-δ OR heteromers requires the expression of a chaperone protein, receptor transport protein-4 (Decaillot et al., 2008). Moreover, the presence of receptor transport protein-4 protects the μ OR-δ OR heteromer from ubiquitination and degradation (Decaillot et al., 2008). Another study showed that morphine and the opioid antagonists naltrexone and naltriben could serve as chemical chaperones that increase the cell surface expression of μ OR-δ OR heteromers (Gupta et al., 2010). With regards to heteromer internalization one study used cells that expressed μ OR and where δ OR expression was induced by treatment with ponasterone A treatment to show that the receptor protomers internalized independently from each other (Law et al., 2005). However, other studies showed that treatment with some μ OR or δ OR agonists (DAMGO, methadone, Deltorphin II, SNC80) but not others (morphine, DPDPE, DSLET) could induce μ OR-δ OR heteromer internalization (Hasbi et al., 2007; He et al., 2011; Milan-Lobo and Whistler, 2011). Interestingly, internalized heteromers are degraded (He et al., 2011; Milan-Lobo and Whistler, 2011) while internalized receptor homomers are recycled to the cell surface (Milan-Lobo and Whistler, 2011). Taken together these studies indicate that μ OR-δ OR heteromers exhibit unique properties compared to individual receptor homomers.

A final and important criteria for a μ OR-δ OR heteromer is the development of unique reagents that selectively target or disrupt the biochemical fingerprint of the heteromer. Several such reagents have been generated including (i) TAT fused peptides that disrupt μ OR-δ OR heteromerization *in vitro* as well as *in vivo* (He et al., 2011; Kabli et al., 2014); (ii) bivalent ligands that are more potent than morphine and without significant development of tolerance and dependence (Daniels et al., 2005); (iii) heteromer-selective antibodies that block μ OR-δ OR heteromer-mediated signaling (Gupta et al., 2010); and (iv) a small molecule μ OR-δ OR biased agonist, CYM51050, that is as potent as morphine but with lower development of tolerance (Gomes et al., 2013c). In the case of TAT fused peptides, a peptide fused to the transmembrane domain 1 of μ OR disrupted the formation of μ OR-δ OR heteromers both in heterologous cells and in the spinal cord (He et al., 2011). Disruption of μ OR-δ OR heteromers in the spinal cord, in turn, led to an increase in morphine-mediated analgesia (He et al., 2011). Another peptide that could disrupt the formation of μ OR-δ OR heteromers in heterologous cells comprised of a TAT peptide fused to a sequence corresponding to the distal carboxyl terminal tail of δ OR (Kabli et al., 2014). Interestingly, intra-accumbens administration of this TAT peptide attenuated the antidepressant and antianxiolytic effects of the δ OR agonist UFP-512 (Kabli et al., 2014). In the case of bivalent ligands a compound comprising a δ OR antagonist that is separated from a μ OR agonist by a 21-atom spacer arm has been synthesized and named MDAN21 (Daniels et al., 2005). Studies show that MDAN21-mediated antinociception is 100 times more potent than that of morphine and that chronic administration of this compound does not lead to the development of tolerance and dependence (Daniels et al., 2005). In addition, MDAN21 prevents the internalization of μ OR-δ OR heteromers probably by occupying both protomers and immobilizing the heteromer at the cell surface (Yekkirala et al., 2013). Other bivalent ligands consisting of high-affinity μ OR ligands (oxymorphone or naltrexone) linked by a spacer arm to low-affinity δ OR ligands (ENTI or DM-SNC80 respectively) have also been synthesized (Harvey et al., 2012); however not much is known about the analgesic effects of these ligands and whether their administration leads to side-effects. In the case of monoclonal μ OR-δ OR heteromer-selective antibodies studies show that they can block the ability of low concentrations of a δ OR selective antagonist, TIPPψ, to potentiate the binding and signaling by DAMGO, a selective μ OR agonist (Gupta et al., 2010). More recently, a small molecule μ OR-δ OR biased agonist, CYM51050, was identified by high-throughput screening of a small molecule library using a β-arrestin recruitment assay (Gomes et al., 2013c). Studies with CYM51010 show that it is more efficacious at activating G-proteins and recruiting β-arrestin in cells expressing the μ OR-δ OR heteromer compared to cells expressing either μ OR or δ OR homomers (Gomes et al., 2013c). In addition, while the antinociceptive activity of CYM51010 is similar to that of morphine, chronic administration of this biased agonist results in lower antinociceptive tolerance compared to morphine (Gomes et al., 2013c). That the signaling and antinociceptive effects of CYM51010 are mostly mediated via μ OR-δ OR heteromers is supported by the observation that they can be partly but significantly blocked by μ OR-δ OR heteromer-selective antibodies (Gomes et al., 2013c). Taken together, these unique heteromer targeting reagents show that μ OR-δ OR heteromers occur *in vivo* and that the heteromer-selective antibodies are useful not only in detecting the presence of an heteromer in endogenous tissue under normal and pathological conditions but also to study the properties of the heteromers and to identify heteromer selective ligands.

## κOR-δOR heteromerization

Localization studies examining heteromerization between δ OR and κ opioid receptors (κ OR) found them to be expressed in the same neuroblastoma cell line (Baumhaker et al., 1993) and co-expressed in axons of the dorsal horn of the spinal cord (Wessendorf and Dooyema, 2001). Co-immunoprecipitation studies using lysates from cells expressing differentially epitope tagged receptors (Jordan and Devi, 1999) or from peripheral sensory neurons (Berg et al., 2012) show that δ OR and κ OR form interacting complexes. That these two receptors are in close proximity for direct receptor-receptor interactions was demonstrated through the use of bioluminescence resonance energy transfer assays (BRET) (Ramsay et al., 2002). Signaling studies in cells co-expressing δ OR and κ OR show a unique “biochemical fingerprint” *in vitro* compared to cells expressing individual receptors (Jordan and Devi, 1999) since they report (i) a decrease in the binding affinity of δ OR or κ OR agonists; (ii) an increase in the binding affinity of a combination of δ OR and κ OR agonists or antagonists; (iii) an increase in signaling with a combination of δ OR and κ OR agonists; and (iv) that etorphine is not able to internalize δ OR; etorphine internalizes δ OR in cells expressing only this receptor (Jordan and Devi, 1999). However, it is not known whether this “biochemical fingerprint” observed for δ OR-κ OR heteromers in heterologous cells co-expressing epitope-tagged receptors is also observed in endogenous tissue. Studies with unique reagents targeting δ OR-κ OR heteromers show that a bivalent ligand, KDN-21, made up of a κ OR antagonist, 5′-GNTI, that is joined by a spacer arm to a δ OR antagonist, naltrindole, exhibits antagonistic activity but has no antinociceptive activity (Bhushan et al., 2004). Another reagent, 6′-guanidinonaltrindole (6′-GNTI) was initially identified as a δ OR-κ OR selective agonist that exhibits antinociceptive activity when administered intrathecally (i.t.) but not intracerebroventricularly (i.c.v) (Waldhoer et al., 2005). However, recent studies have reported that 6′-GNTI exhibits biased agonistic activity for κ OR both in heterologous cells and striatal neurons (Rives et al., 2012; Schmid et al., 2013). This brings into question the selectivity of this compound for δ OR-κ OR heteromers. Finally, a δ OR-κ OR heteromer selective antibody has been generated and characterized (Table 1). Although not much is known about the ability of this antibody to block heteromer-mediated binding and signaling, it has been useful in demonstrating a role for δ OR-κ OR heteromer function in *vivo* (Berg et al., 2012). Administration of the δ OR-κ OR heteromer selective antibody into the hind paw of rats potentiated the antinociceptive response of a subthreshold dose of DPDPE such that the latter now gave nearly the maximal possible antinociceptive response required to inhibit the thermal allodynia produced by PGE2 (Berg et al., 2012). Since, treatment with the κ OR antagonist, nor-BNI, also increases the antinociceptive response of a subthreshold dose of DPDPE although not to the same extent as the δ OR-κ OR heteromer selective antibody (Berg et al., 2012), these results suggest that either drugs targeting the δ OR-κ OR heteromer or a combination of the heteromer-selective antibody with DPDPE would be more effective in the treatment of thermal allodynia.

## δOR-CB1R heteromerization

A number of early studies suggested interactions between δ OR and CB1 cannabinoid receptors (CB1R). These included (i) additive effects on signaling in N18TG neuroblastoma cells by a combination of opioid and cannabinoid ligands (Shapira et al., 1998); (ii) release of leucine-enkephalin during Δ^9^-THC-mediated antinociception (Welch and Eads, 1999); (iii) signal cross-desensitization between CB1R and δ OR (Shapira et al., 1998); (iv) the anxiolytic effects of the CB1R agonist, Δ^9^-THC, could be blocked by the δ OR antagonist, naltrindole (Berrendero and Maldonado, 2002); (v) increases in the levels and activity of CB1R in some brain regions of δ OR knockout mice (Berrendero et al., 2003); and (vi) increases in δ OR activity in CB1R knockout mice (Uriguen et al., 2005). Co-localization studies demonstrated the presence of CB1R and δ OR in the same neuroblastoma cell line (Shapira et al., 1998) and within the cell bodies and processes of primary cortical neurons (Rozenfeld et al., 2012). Moreover, co-immunoprecipitation studies detect the formation of interacting CB1R-δ OR complexes only in cells that co-express both receptors (Rozenfeld et al., 2012) and proximity based assays show that both receptors are in close proximity for direct receptor-receptor interactions in live cells (Rios et al., 2006). Examination of unique signaling showed that the δ OR-CB1R heteromer exhibits a distinct biochemical fingerprint in heterologous cells in that (i) the presence of δ OR or low concentrations of δ OR ligands decreases the signaling potency of a CB1R agonist in heterologous cells and this is not seen in cells with a knockdown of δ OR levels (Rozenfeld et al., 2012); (ii) the activity of CB1R is increased in cortical membranes from δ OR knockout mice (Rozenfeld et al., 2012); (iii) in the presence of δ OR a CB1R agonist activates a pathway involving phospholipase C (PLC) and β-arrestin2 while in the absence of δ OR it activates Gαi/o-mediated signaling (Rozenfeld et al., 2012); (iv) in cells co-expressing CB1R and δ OR activation of CB1R leads to accumulation of phosphorylated ERk1/2 in centrosomes (Rozenfeld et al., 2012); (v) activation of CB1R promotes increased cell survival only in cells co-expressing CB1R and δ OR (Rozenfeld et al., 2012); and (vi) treatment with a CB1R antagonist decreases the survival of primary cortical neurons from wild-type but not from δ OR knockout mice (Rozenfeld et al., 2012). Additional studies supporting heteromerization between δ OR and CB1R include those examining the maturation and trafficking of the two receptors. These studies show that co-expression of δ OR changes the localization of CB1R from an intracellular compartment to the cell surface and this involves increased association with the adaptor protein AP-2 (Rozenfeld et al., 2012) and, reducing δ OR levels in F11 cells that co-express CB1R and δ OR leads to a decrease in the surface expression of CB1R (Rozenfeld et al., 2012).

To date reagents that selectively disrupt the δ OR-CB1R heteromer or ligands targeting this heteromer have not been developed. However, antibodies that selectively recognize δ OR-CB1R heteromers have been generated and characterized (Table 1). The δ OR-CB1R heteromer-selective antibody was used to examine the regulation of the heteromer during neuropathic pain. We detected changes in heteromer levels 14 days after induction of neuropathic pain. Specifically, the antibody detected significant increases in δ OR-CB1R heteromer levels in cortex, hypothalamus and midbrain of animals exhibiting neuropathic pain (Bushlin et al., 2012). This antibody was also useful in determining the heteromer-selective fingerprint in that it could block CB1R agonist-mediated increases in δ OR activity and this was seen only in membranes from animals with neuropathic pain (Bushlin et al., 2012). Taken together, these studies indicate that the δ OR-CB1R heteromer could be a novel therapeutic target in the treatment of neuropathic pain. Moreover, the δ OR-CB1R heteromer-selective antibody could also be a potential therapeutic for the treatment of neuropathic pain given that it could block heteromer- mediated signaling.

## CB1R-AT1R heteromerization

Studies showing an increase in CB1R levels in liver cells that also express AT1 angiotensin receptors (AT1R) suggested possible interactions between these receptors (Teixeira-Clerc et al., 2006; Mallat and Lotersztajn, 2008; Siegmund and Schwabe, 2008; Lanthier et al., 2009). Co-localization of CB1R with AT1R has been demonstrated in hepatic stellate cells activated in response to chronic ethanol administration (Rozenfeld et al., 2011). Furthermore, co-immunoprecipitation studies show that CB1R and AT1R form interacting complexes in these cells (Rozenfeld et al., 2011). Examination of the biochemical profile of the CB1R-AT1R heteromer shows that the AT1R agonist induces a rapid and robust increase in ERK1/2 phosphorylation via G_α i_ instead of G_α q_ in cells co-expressing both receptors, and this is reduced either by decreasing the levels of CB1R using siRNA, or by inhibiting the activity of diacylglycerol lipase, the enzyme involved in the synthesis of the endocannabinoid 2-arachidonoylglycerol (Rozenfeld et al., 2011). In addition, CB1R ligands modulate AT1R-mediated increases in ERK1/2 phosphorylation with agonists potentiating and antagonists blocking signaling (Rozenfeld et al., 2011). Moreover, in cells co-expressing CB1R and AT1R phosphorylation of ERK1/2 by a CB1R agonist is only detected in the presence of a very low non-signaling dose of an AT1R agonist (Rozenfeld et al., 2011). Another interesting feature of cells co-expressing CB1R and AT1R is that although CB1R activation does not lead to increases in intracellular Ca^+2^ levels, activation of AT1R induces increases via G_α q_ but this requires the presence of CB1R since it is attenuated following siRNA-mediated knockdown of CB1R (Rozenfeld et al., 2011). Additional support for direct interactions between CB1R and AT1R comes from studies examining the maturation of these receptors. These studies show that the expression of AT1R changes the localization of CB1R from an intracellular compartment to the plasma membrane in Neuro 2A cells (Rozenfeld et al., 2011).

Although reagents that selectively disrupt CB1R-AT1R heteromers and ligands that selectively target this heteromer pair have not as yet been generated, antibodies that selectively recognize this heteromer have (Table 1). The CB1R-AT1R heteromer-selective antibody was used to examine the heteromer signaling fingerprint. The antibody can block angiotensin II-mediated ERK1/2 phosphorylation only in cells expressing the heteromer but not when CB1R levels are reduced in these cells by siRNA treatment; this indicates that ERK1/2 phosphorylation by angiotensin II is mediated via the CB1R-AT1R heteromer (Rozenfeld et al., 2011). In addition, the antibody can block the secretion of fibrogenic proteins including α-SMA from activated hepatic stellate cells obtained from rats chronically treated with ethanol (Rozenfeld et al., 2011). This together with observations indicating that the profibrogenic activity of AT1R in ethanol induced liver fibrosis requires the presence of CB1R (Rozenfeld et al., 2011) suggest that the CB1R-AT1R heteromer represents a novel therapeutic target for the treatment of liver fibrosis and that the CB1R-AT1R heteromer by its ability to block the secretion of fibrogenic proteins could potentially be used as a therapeutic to treat liver fibrosis.

## Conclusions

In this review we describe how a subtractive immunization strategy can be successfully used to generate monoclonal antibodies that are selective for a given heteromer pair and that can be useful for examination of endogenous heteromers. Even though the procedure is time consuming, and requires a number of controls during screening procedures for determining the heteromer-selectivity of the antibodies, there are many advantages to developing the heteromer selective antibodies. These include the fact that they recognize a unique epitope that is present only in cells/tissues expressing the heteromer of interest, and thus they could be used to map the targeted heteromer in endogenous tissue and to monitor changes in heteromer levels during pathological conditions. In addition, heteromer-selective antibodies are also useful to discriminate the contribution of the heteromer from individual receptor homomers for a given signaling response. Finally, in select cases heteromer-selective antibodies have been useful to block a biological response. In this case the antibodies could be used as therapeutic targets in pathological conditions where heteromer levels are upregulated in addition to being useful in the identification of heteromer-selective/biased ligands. Thus, heteromer-selective antibodies represent unique and invaluable tools that would help in our understanding of the physiological roles of GPCR heteromers in endogenous tissues.

### Conflict of interest statement

The authors declare that the research was conducted in the absence of any commercial or financial relationships that could be construed as a potential conflict of interest.
